# Developing PspCas13b-based enhanced RESCUE system, eRESCUE, with efficient RNA base editing

**DOI:** 10.1186/s12964-021-00716-z

**Published:** 2021-08-11

**Authors:** Guo Li, Yihan Wang, Xiangyang Li, Yuzhe Wang, Xingxu Huang, Jianen Gao, Xiaoxiang Hu

**Affiliations:** 1 State Key Laboratory of Agrobiotechnology, China Agricultural University, 2 Yuanmingyuan West Road, Haidian District, Beijing, 100193 China; 2 College of Biological Sciences, China Agricultural University, 2 Yuanmingyuan West Road, Haidian District, Beijing, 100193 China; 3 National Research Institute for Family Planning, 12 Dahuisi Road, Haidian District, Beijing, 100081 China; 4 Graduate School of Peking, Union Medical College, 9 Dongdan Santiao, Dongcheng District, Beijing, 100730 China; 5 School of Life Science and Technology, ShanghaiTech University, 100 Haike Road, Pudong New Area, Shanghai, 200031 China; 6 College of Animal Science and Technology, China Agricultural University, 2 Yuanmingyuan West Road, Haidian District, Beijing, 100193 China; 7 CAS Center for Excellence in Molecular Cell Science, Shanghai Institute of Biochemistry and Cell Biology, Chinese Academy of Sciences, University of Chinese Academy of Sciences, 320 Yueyang Road, Shanghai, 200031 China

**Keywords:** RNA base editing, eRESCUE, Phosphorylation, *IK*Kβ

## Abstract

RNA base editing is potential for cellular function research and genetic diseases treating. There are two main RNA base editors, REPAIR and RESCUE, for in vitro use. REPAIR was developed by fusing inactivated Cas13 (dCas13) with the adenine deaminase domain of ADAR2, which efficiently performs adenosine-to-inosine (A-to-I) RNA editing. RESCUE, which performs both cytidine-to-uridine (C-to-U) and A-to-I RNA editing, was developed by fusing inactivated Cas13 (dCas13) with the evolved ADAR2. However, the relatively low editing efficiency of the RESCUE system limits its broad application. Here, we constructed an enhanced RESCUE (eRESCUE) system; this dPspCas13b-RESCUE-NES system was generated by fusing inactivated PspCas13b with the evolved ADAR2. We determined the endogenous mRNA A-to-I and C-to-U editing efficiency mediated by the dPspCas13b-RESCUE-NES system in HEK-293T cells. This new RNA base editor was then used to induce 177Ser/Gly conversion of inhibitor kappa B kinase β (*IKKβ*) by changing the genetic code from AGU to GGU. The results showed that the eRESCUE editor mediates more efficient A-to-I and C-to-U RNA editing than the RESCUE RNA editor, as was previously reported. The 177Ser/Gly conversion of *IKKβ*, accomplished by converting the genetic code from AGU to GGU, resulted in a decrease in the phosphorylation of *IK*Kβ and downregulation of downstream *IKKβ*-related genes. In summary, we developed a more efficient RNA base editor, eRESCUE, which may provide a useful tool for biomedical research and genetic disease treatment.

**Video Abstract**

**Supplementary Information:**

The online version contains supplementary material available at 10.1186/s12964-021-00716-z.

## Background

Previous studies have reported several tools that mediate adenosine-to-inosine (A-to-I) RNA editing in vivo [1–3]. Currently, there are two main RNA base editing tools in vitro: RNA editing for programmable A-to-I replacement (REPAIR) [4] and RNA editing for specific C-to-U exchange (RESCUE) [5]. REPAIR was the first RNA base editor developed and was constructed using a catalytically inactivated Cas13 ortholog from *Prevotella sp.* (dPspCas13b) fused with the adenosine deaminase acting on RNA type 2 (ADAR2) [4]. To expand the application of RNA editor systems, a RESCUE RNA base editor that performs both C-to-U and A-to-I RNA editing was successfully developed by fused with the evolved ADAR2 which served as a cytidine deaminase, then fused to an inactivated Cas13 ortholog from *Riemerella anatipestifer* (dRanCas13b) [5]. However, it should be noted that the efficiency of both A-to-I and C-to-U RNA editing is relatively low, especially for C-to-U base editing.

In this study, we tried to improve the RNA editing efficiency of the RESCUE system by using a Cas13 ortholog, dPspCas13b, with a nuclear export sequence (NES) to guide RNA base editors to edit mRNA in the cytoplasm. We successfully developed a PspCas13b-based enhanced RESCUE system, eRESCUE, with efficient RNA base editing abilities.

## Methods

### Design and cloning of mammalian constructs for RNA editing

Several plasmids, including pC0078 RESCUE (#130661) [5], containing dRanCas13b-ADAR2dd (RESCUE) with a C-terminal fusion of mapk NES (dRanCas13b-RESCUE-NES), and the pC0043-PspCas13b crRNA backbone (#103854) [5]. This contains a 3′ direct repeat and can be cloned using BbsI sites. pC0039-CMV-dPspCas13b-GS-ADAR2DD (E488Q) (#103849) [4] was purchased from Addgene (https://www.addgene.org/). RNA editing sites and the sgRNA sequences could be found in Additional file 1: Table S1. To construct the dPspCas13b-RESCUE-NES expression vector, the dPspCas13b fragment was amplified using the pC0039-CMV-dPspCas13b-GS-ADAR2DD(E488Q) plasmid as a template and cloned into the pC0078 RESCUE plasmid, in which the dRanCas13b fragment was removed. The RNA base editors' specific sequences could be found in Additional file 1: Table S4.

### Cell culture

293T cells were cultured in Dulbecco’s modified Eagle’s medium (Gibco) with 10% foetal bovine serum (FBS) (v/v) (Gemini), and maintained at 37 °C with 5% CO_2_ under standard humidity conditions.

### RESCUE editing in mammalian cells

Before transfection, 293T cells were seeded in 24-well plates which have been coated by D-lysine, and maintained at approximately 60–70% confluence. Then cells were transfected using EZ Trans Reagent (Shanghai Life iLab) according to the manufacturer’s protocols. For transfection, dPspCas13b-RESCUE or the dRanCas13b-RESCUE- (800 ng) and gRNA-expressing plasmids (400 ng) were mixed and added to each well. DNA (1.2 µg) and 3.6 µL EZ Trans Reagent were diluted in 50 µL DMEM. The diluted EZ Trans Reagent was then added into the diluted DNA solution, mixed gently, and incubated for 15 min at room temperature (20–25 °C) to form DNA-EZ Trans Reagent complexes. After 15 min of incubation, the DNA-EZ Trans Reagent complexes were directly added to each well and mixed gently by rocking the plate back and forth. At 6 h post-transfection, the complexes were removed, and 0.5 mL complete growth medium was added to the cells. Post-transfection 48 h, GFP-positive cells were collected by fluorescence-activated cell sorting (FACS).

### Flow cytometry

Cells were collected and subjected to FACS at 48 h after transfection. The GFP signal was detected via FACS. Cells (2 × 10^4^) with positive GFP signals were collected and used to extract total RNA for editing efficiency analysis. More than 5 × 10^5^ GFP-positive cells were harvested and used to extract total RNA for off-target analysis.

### RNA editing efficiency analysis

GFP-positive cells were sorted by FACS. Total RNA of the collected cells was immediately extracted by using the TRIzol reagent (Invitrogen) according to the manufacturer’s instructions. cDNA was generated by using the HiScript II Q RT SuperMix (Vazyme). Phanta® Max Super-Fidelity DNA Polymerase (Vazyme) was used for PCR amplifying. The PCR amplification primers are listed in Additional file 1: Table S2. The online software EditR (https://moriaritylab.shinyapps.io/editr_v10/) was used to calculate editing efficiency by analysing the Sanger sequencing results of the PCR-amplified fragments.

### Immunofluorescence analysis

Briefly, cells were fixed in 4% (w/v) paraformaldehyde (SIGMA-ALDRICH, 158127) for 10 min at room temperature (20–25℃), permeabilized, and blocked for 30 min with 1% (w/v) bovine serum albumin (SIGMA-ALDRICH, V900933), and then permeabilized with 0.1% (v/v) Triton X-100. Fixed cells were washed and incubated with a primary antibody (ab16502) at 4 °C overnight. The cells were then incubated with a secondary antibody, goat anti-rabbit IgG H&L (HRP) (abcam: # ab97051) for 2 h, and the nuclei were stained with DAPI. TRITC-phalloidin staining was used to visualise the cytoskeleton. Imaging was performed by confocal microscopy (Nikon A1R).

### Differential gene expression analysis

Total RNA from GFP-positive cells was extracted using TRIzol reagent. cDNA was synthesised using oligo d (T) primers and used as the templates for real-time PCR. SYBR green-based real-time PCR was performed to evaluate the mRNA expression level. PCR primers were showed in Additional file 1: Table S3. The reaction was performed at 95 °C for 2 min, followed by 40 cycles of 95 °C for 15 s and 61 °C for 1 min using an ABI 7300 detection system. The standard curve method was used for quantification, and the cDNA of the detected mRNA was tenfold serially diluted to generate the standard curve. The mRNA quantities of the samples were determined by linear extrapolation of the Ct values plotted against the standard curve. All assays were repeated at least three times, and each experiment was performed in triplicate. One-way or two-way ANOVA with multiple comparison corrections was used to assess the statistical significance of transcript changes using Prism 7.

### Whole-transcriptome sequencing

To examine RNA off-target effects across the transcriptome, each selected sample was comprised of two biological repeats, and total RNA was extracted using TRIzol. A total amount of 1 µg RNA per sample was used as input material for the RNA sample preparations. Sequencing libraries were generated using NEBNext® UltraTM RNA Library Prep Kit for Illumina® (NEB, USA) following manufacturer’s recommendations and index codes were added to attribute sequences to each sample. All the cDNA samples were sequenced by Novogene Co., Ltd.

GATK2 (v3.7) software was used to perform SNP calling. Raw vcf files were filtered with GATK standard filter method and other parameters (cluster:3; WindowSize:35; QD < 2.0 o; FS > 30.0; DP < 10 and Snp Eff software was used to annotation for the variable site.

### Statistical analysis

All data are presented as mean ± SEM, as indicated. Statistical analysis of the results was performed using GraphPad Prism (GraphPad PRISM, Version 8.0). The statistical difference between the two groups was evaluated using one-way ANOVA. All data are expressed as arithmetic mean ± SEM. The level of significance was set at *p* < 0.05, whereas asterisks show differences at the following levels: *p < 0.05, **p < 0.01, ***p < 0.001.

## Results

### dPspCas13b-RESCUE-NES performed improved RNA base editing at exogenous sites relative to dRanCas13b-RESCUE-NES

**Fig. 1:**
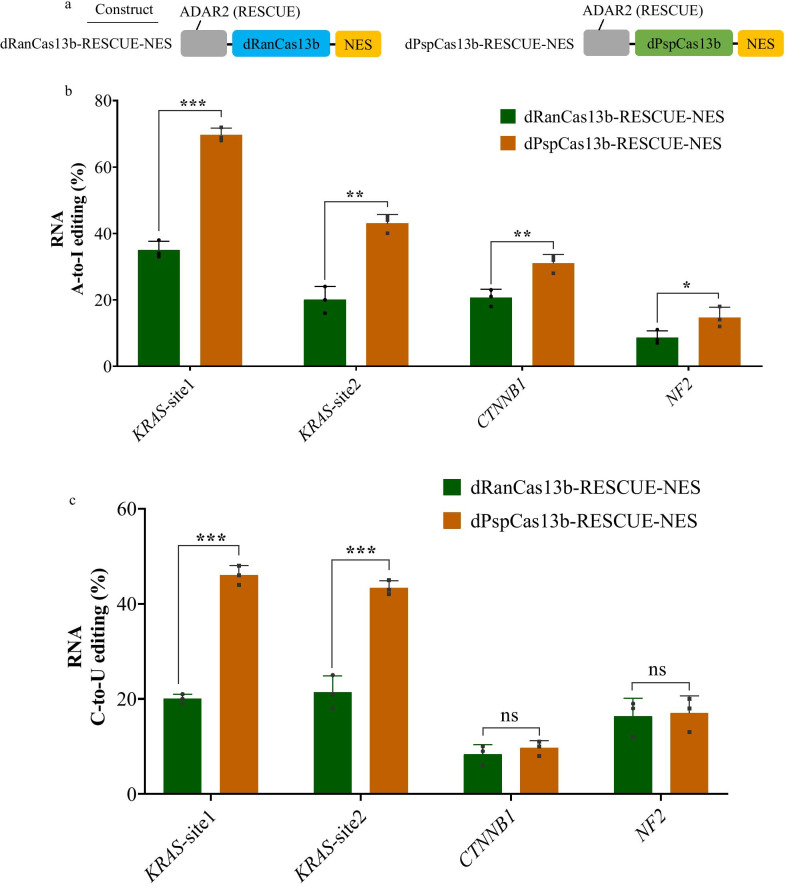
dPspCas13b-RESCUE and dRanCas13b-RESCUE systems mediated exogenous mRNA A-to-I or C-to-U editing in 293T cells. **a** Schematic representative of the engineered dPsp/dRanCas13b-RESCUE system. **b** A-to-I RNA editing efficiency analysis of NES-guided dPsp/dRanCas13b-RESCUE systems. Bars represent the mean ± SEM. Different asterisks represent significant differences as follows: *p < 0.05; **p < 0.01; ***p < 0.001. **c** C-to-U RNA editing efficiency analysis of NES-guided dPsp/dRanCas13b-RESCUE systems. Bars represent the mean ± SEM. “***”*p* < 0.001; ns, no significance

In eukaryotic cells, the primary transcript (pre-mRNA) is synthesised from a DNA template in the cell nucleus by transcription, and the pre-mRNA is completely processed to mature messenger RNA (mRNA) in the cytoplasm [6]. The dRanCas13b protein is guided by the NES for mRNA A-to-I and C-to-U editing in the RESCUE system. Given that PspCas13b mediated the highest efficiency in knocking down endogenous *KRAS* compared to other optimised Cas13b systems or hairpin-mediated RNA (shRNA) [4], we generated mammalian codon-optimized dPspCas13b and constructed the dPspCas13b-based RESCUE system by replacing dRanCas13b with dPspCas13b to explore whether the dPspCas13b-RESCUE system could be more efficient. Two RESCUE RNA base editors, dPspCas13b-RESCUE-NES and dRanCas13b-RESCUE-NES, were constructed (Fig. 1a). We selected four targets, *KRAS* site 1 and 2, *CTNNB1*, and *NF2*, which showed relatively high A-to-I or C-to-U editing efficiency in the dRanCas13b-RESCUE system [5]. We compared the A-to-I and C-to-U RNA editing efficiency in 293T cells by co-transfection with the dPspCas13b-RESCUE or dRanCas13b-RESCUE systems with the sgRNA expression constructs for target sites (i.e., *KRAS* site 1 and site 2, *CTNNB1*, and *NF2)*. At 48 h post-transfection, we collected 2 × 10^4^ GFP-positive cells by FACS. We extracted the total RNA of the 2 × 10^4^ GFP-positive cells and obtained cDNA by reverse transcription. The targeted sequences were amplified by PCR and subjected to Sanger sequencing. We used the general analytic tool, EditR (https://moriaritylab.shinyapps.io/editr_v10/) [7] to analyse the Sanger sequencing results for calculating the editing efficiency. Notably, the results showed that the dPspCas13b-RESCUE-NES system mediated A-to-I RNA editing that is more efficient than the dRanCas13b-RESCUE-NES system. In particular, the A-to-I RNA editing efficiency of *KRAS* site 1 mediated by dPspCas13b-RESCUE-NES was up to 78% (Fig. 1b).

Similarly, the dPspCas13b-RESCUE-NES system showed more efficient C-to-U RNA editing than the dRan-RESCUE-NES system at two sites, *KRAS* site 1 and 2 (Fig. 1c). As shown in Fig. 1c, the C-to-U RNA editing efficiency of *KRAS* site 2 mediated by dPspCas13b-RESCUE-NES was up to 58%. The results demonstrated that the NES guided dPspCas13b-RESCUE system might be a more efficient RNA editor for RNA A-to-I and C-to-U editing.

### dPspCas13b-RESCUE-NES performed better RNA editing at endogenous sites than dRanCas13b-RESCUE-NES

**Fig. 2:**
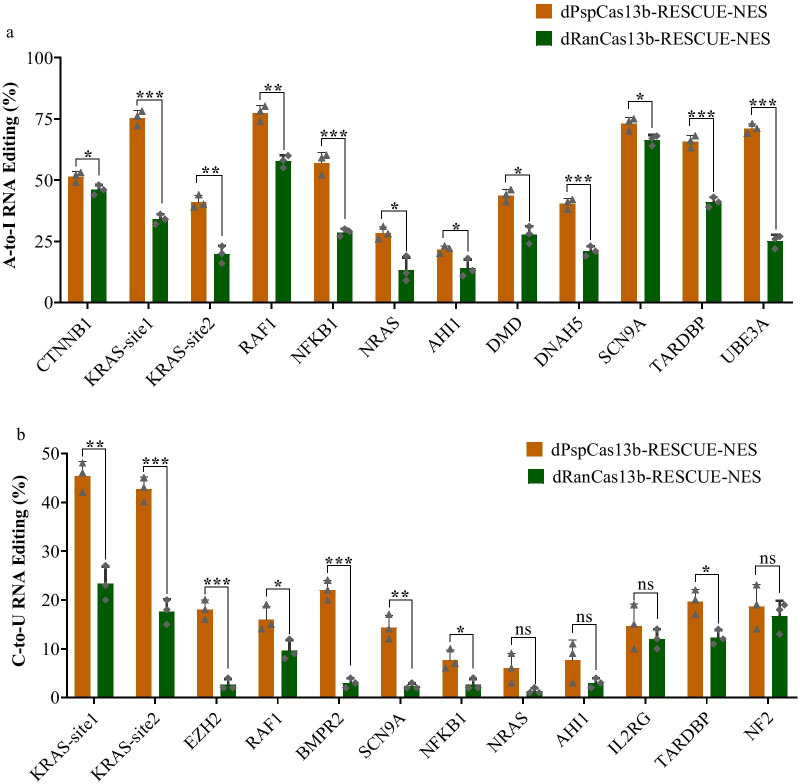
dPsp-RESCUE-NES system mediated efficient endogenous mRNA A-to-I or C-to-U editing in 293T cells. **a** The comparison of A-to-I RNA editing efficiency between the dPspCas13b-RESCUE-NES and dRanCas13b-RESCUE-NES systems. Bars represent the mean ± SEM. Different asterisks indicate significant differences as follows: *p < 0.05; **p < 0.01; ***p < 0.001. **b** The comparison of C-to-U RNA editing efficiency between dPspCas13b-RESCUE-NES and dRanCas13b-RESCUE-NES systems. Bars represent the mean ± SEM. Different asterisks indicate significant differences as follows: *p < 0.05; **p < 0.01; ***p < 0.001; ns, no significance

To further characterise the NES guided RESCUE RNA editors, dPspCas13b-RESCUE-NES, and dRanCas13b-RESCUE-NES were used to target more endogenous sites. A total of 12 transcripts of endogenous sites, including *CTNNB1*, *KRAS* site 1 and 2, *RAF1*, *NFKB1*, *NRAS*, *AHI1*, *DMD*, *DNAH5*, *SCN9A*, *TARDBP*, and *UBE3A*, were selected to compare the A-to-I mRNA base editing efficiency between the dPspCas13b-RESCUE-NES and dRanCas13b-RESCUE-NES system. In parallel, a total of 12 endogenous site transcripts, including *KRAS* site 1 and 2, *EZH2*, *RAF1*, *BMPR2*, *SCN9A*, *NFKB1*, *NRAS*, *AHI1*, *IL2RG*, *TARDBP*, and *NF2*, were selected to compare the C-to-U mRNA base editing efficiency between the two Cas13b-RESCUE systems. The results showed that the dPspCas13b-RESCUE-NES system mediated higher A-to-I editing efficiency than dRanCas13b-RESCUE-NES system in all 12 endogenic sites, including *CTNNB1*, *KRAS* site 1, *KRAS* site 2, *RAF1*, *NFKB1*, *NRAS*, *AHI1*, *DMD*, *DNAH5*, *SCN9A*, *TARDBP*, and *UBE3A* (Fig. 2a). In addition, the dPspCas13b-RESCUE-NES system mediated higher C-to-U editing efficiency than the dRanCas13b-RESCUE-NES system for transcripts of eight endogenic sites, including *KRAS* site 1, *KRAS* site 2, *EZH2*, *RAF1*, *BMPR2*, *SCN9A*, *NFKB1*, and *TARDBP* (Fig. 2b). Taken together, the dPspCas13b-RESCUE-NES system functioned substantially better at mediating endogenous mRNA base editing compared to the dRanCas13b-RESCUE-NES system.

### *IKKβ* 177Ser/Gly substitution via dPspCas13b-RESCUE-NES system resulted in AGU/GGU genetic code change

**Fig. 3:**
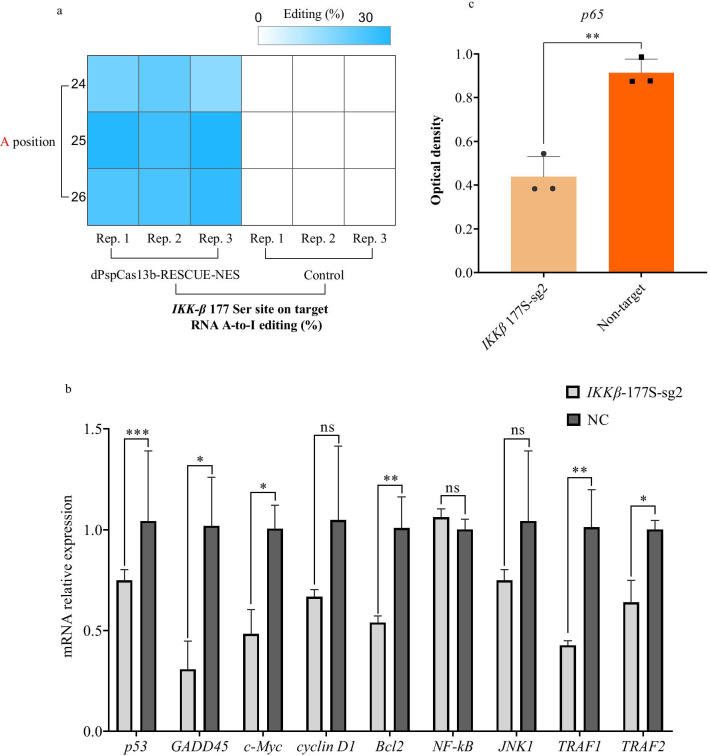
Substitution of *IKKβ* 177Ser/Gly mediated by dPspCas13b-RESCUE-NES system. **a** Heatmap of the A-to-I editing rate on adenosines covered by dPspCas13b-RESCUE-NES system targeting the *IKKβ* mRNA. Three gRNAs that target A and are positioned at 24, 25, and 26 were designed. Three replicates were performed per gRNA for A-to-I editing efficiency statistics. **b** The effective editing of targeted *IKKβ* mRNA contributed to *IKKβ*-related gene expression detection. Bars represent the mean ± SEM. Different asterisks indicate significant differences as follows: *p < 0.05; **p < 0.01; ***p < 0.001; ns, no significance. **c** The red optical density statistics of p65 proteins in the cell nucleus. Statistical method: The analysis tool Image J was used to determine the grey scale of the mCherry protein located in the nucleus of cells in the IKKβ-177 Ser gRNA group and the non-target control group for semiquantitative analysis. Three representative pictures from each group were analysed. Bars represent mean ± SEM. “**”*p* < 0.01

Given the success of the dPspCas13b-RESCUE-NES RNA base editor in vitro, it was next examined in biological studies. Diverse cellular stresses, such as inflammatory cytokines, bacterial or viral products and DNA damage could activate the *NF-κB* signalling pathway [8]. Genes involved in immune response, growth control and protection against apoptosis are activated by unbound *NF-κB,* which located in the nucleus. *NF-κB* activation depends on the *IκB* kinase (*IKK*). The phosphorylation of various *IκB* and *NF-κB* proteins are catalysed by *IKK* complex integrates signals which are from *NF-κB* activating stimuli [9]. Phosphorylation of Ser 177 and Ser 181 which locate in the activation loop of *IKKβ* determine the activation of *IKK* [10]. Taking advantage of dPspCas13b-RESCUE-NES, we set out to study the effects of phosphorylation of 177 Ser of *IKKβ*. We designed three dPspCas13b-RESCUE-NES guides for *IKKβ* 177Ser (AGU)/Gly (GGU) substitution by mediating *IKKβ* mRNA A-to-I editing. We constructed three sgRNA expression vectors in which the target base A is positioned at location 24, 25, and 26 for *IKKβ* 177Ser (AGU)/Gly (GGU) substitution. We then detected the A-to-I efficiency of the three sgRNAs that were mediated by the dPspCas13b-RESCUE-NES system in 293T cells. The results showed that the A-to-I editing efficiency of three replicates were 23%, 26%, and 19% for position 24 of sgRNA1; 36%, 32%, and 37% for position 25 of sgRNA2; and 29%, 30%, 34%, for position 26 of sgRNA3, respectively (Fig. 3a). Then, we used sgRNA2, which performed best among the three sgRNAs for *IKKβ* mRNA A-to-I editing.

The sgRNA2 expression construct and dPspCas13b-RESCUE-NES editor were co-transfected into 293T cells. We then performed immunostaining of the *p65* subunit, which is a major component of *NF-κB* complexes and is responsible for trans-activation [11]. The results showed that more *p65* proteins shifted into the cytoplasm in the sgRNA2 and dPspCas13b-RESCUE-NES co-transfection groups compared to that with the control group or the non-targeting sgRNA and dPspCas13b-RESCUE-NES co-transfection groups (Fig. 3c). Consistently, detection of the relative expression of the downstream regulated genes by qPCR showed that the expression of *p53* and *GADD45*, which function in cell cycle regulation, *c-Myc* and *Bcl2* for cell proliferation, and *TRAF1* and *TRAF2* for apoptosis were significantly decreased (Fig. 3b). These results demonstrated that the dPspCas13b-RESCUE-NES system mediated efficient A-to-I base editing, which resulted in the dephosphorylation of *IKKβ* 177Ser by changing the genetic code from AGU to GGU. The dephosphorylation of *IKKβ* 177Ser further downregulated *IKKβ*-related genes. These results indicate that the dPspCas13b-RESCUE-NES RNA editor is a versatile tool for regulation of protein phosphorylation and functional amino acid mutations.

### Examination of RNA off-target effects resulting from the dPspCas13b-RESCUE-NES and dRanCas13b-RESCUE-NES systems

**Fig. 4:**
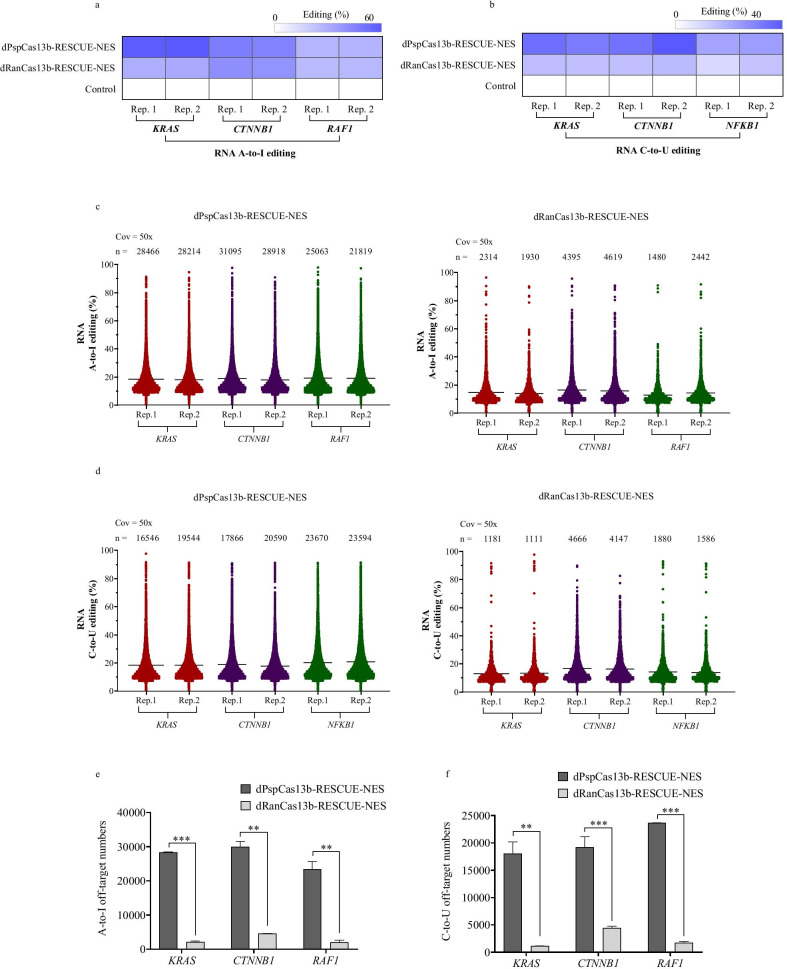
Transcriptome-wide off-target determination with the dPspCas13b-RESCUE-NES and dRanCas13b-RESCUE-NES systems. **a** Heatmap of the A-to-I editing rate of adenines covered by dPspCas13b-RESCUE-NES and dRanCas13b-RESCUE-NES systems targeting *KRAS*, *CTNNB1*, and *RAF1* genes. Each target contains two experimental repeats. **b** Heatmap of the C-to-U editing rate on cytosine covered by dPspCas13b-RESCUE-NES and dRanCas13b-RESCUE-NES system targeting at *KRAS*, *CTNNB1*, and *NFKB1* gene. Each target contains two experiment repeats. **c** Manhattan plots of dPspCas13b-RESCUE-NES and dRanCas13b-RESCUE-NES A-to-I off-targets. n, total number of A-to-I off-target SNPs. **d** Manhattan plots of dPspCas13b-RESCUE-NES and dRanCas13b-RESCUE-NES C-to-U off-targets. n, total number of C-to-U off-target SNPs. **e** Comparison of A-to-I RNA off-target numbers between dPspCas13b-RESCUE-NES and dRanCas13b-RESCUE-NES systems. Bars represent the mean ± SEM. Different asterisks indicate significant differences as follows: **p < 0.01; ***p < 0.001. **f** The comparison of C-to-U RNA off-target numbers between dPspCas13b-RESCUE-NES and dRanCas13b-RESCUE-NES systems. Bars represent the mean ± SEM. Different asterisks indicate significant differences as follows: **p < 0.01; ***p < 0.001

Given that RNA editors usually induce off-target effects, we checked the possible off-targets resulting from the dPspCas13b-RESCUE-NES and dRanCas13b-RESCUE-NES systems at three endogenous sites, including *KRAS* site 1, *CTNNB1*, and *RAF1*. The RNA base editing efficiency of *KRAS* site 1, *CTNNB1*, and *RAF1*, which are mediated by dPspCas13b-RESCUE-NES and dRanCas13b-RESCUE-NES systems were detected. The results showed that the A-to-I on-target efficiency of the dPspCas13b-RESCUE-NES system were 68% and 72% for *KRAS*, 53% and 51% for *CTNNB1*, and 30% and 31% for *RAF1*, respectively; additionally, the dRanCas13b-RESCUE-NES system results were 35% and 40% for *KRAS*, 44% and 46% for *CTNNB1*, and 28% and 29% for *RAF1*, respectively (Fig. 4a). The C-to-U on-target editing efficiency of the dPspCas13b-RESCUE-NES system was 46% and 42% for *KRAS*, 45% and 53% for *CTNNB1*, 29% and 31% for *RAF1*, respectively, whereas the dRanCas13b-RESCUE-NES system were 20% and 21% for *KRAS*, 20% and 22% for *CTNNB1*, and 19% and 12% for *RAF1*, respectively (Fig. 4b). For A-to-I and C-to-U RNA off-target detection, three experimental groups were examined with two replicates per transfection group: dPspCas13b-RESCUE-NES and target sgRNAs group; dRanCas13b-RESCUE-NES group; and target sgRNAs and EGFP expression plasmid transfection groups. Approximately 5 × 10^6^ GFP^+^ positive cells were collected by FACS, and total RNA was extracted from these cells for RNA-seq analysis. We evaluated transcriptome-wide off-targets by RNA-seq over all mRNAs with 50× coverage. Data from the dPspCas13b-RESCUE and target sgRNA transfection groups, as well as the dRanCas13b-RESCUE-NES and target sgRNA transfection groups were filtered by the data of the EGFP expression plasmid transfection groups. We found that there were substantial A-to-I off-target events (Fig. 4c, e) and C-to-U off-target events (Fig. 4d, f). A greater number of A-to-I and C-to-U off-target events appeared with the dPspCas13b-RESCUE-NES RNA editor, suggesting that further optimisation is necessary to decrease the off-targets of the dPspCas13b-RESCUE-NES system.

## Discussion

RESCUE is a potential RNA base editing technology which can mediate both A-to-I and C-to-U conversion in RNA [5]. However, the relatively low editing efficiency of the RESCUE system limits its applications. To improve this system, we comprehensively compared the A-to-I and C-to-U editing efficiency between the two editors, dPspCas13b-RESCUE-NES, and dRanCas13b-RESCUE-NES. Our results demonstrated that dPspCas13b-RESCUE-NES system was more efficient of the two. The dPspCas13b-RESCUE-NES system mediated up to 78% A-to-I editing efficiency (Fig. 2a) and 58% C-to-U editing efficiency (Fig. 2b)—much higher values than that found with previously described RNA editors [4, 5]. These results differ from the results reported in another paper on the RESCUE system by Abudayyeh et al. [5], in which dPspCas13b mediated similar RNA editing efficiency to the dRanCas13b system in yeast. One possible explanation for this difference is that the activity of the dPspCas13b-RESCUE-NES system in mammalian cells may be different from that in yeast. The A-to-I and C-to-U RNA editing efficiency of dRanCas13b-RESCUE-NES system are considerably higher than found in the prior RESCUE study [5], possibly, because we analysed the FACS-sorted positive cells.

To demonstrate the versatility of RNA editing, we applied the more efficient RNA editor, dPspCas13b-RESCUE-NES, to alter the genetic code from AGU to GGU for *IKKβ,* thereby mediating the conversion of site 177 from Ser to Gly, allowing us to explore the impact of *IKKβ* dephosphorylation. As expected, our results showed that more *p65* proteins were arrested in the cytoplasm (Fig. 3c). Consequently, several *IKKβ-*regulated genes related to cell proliferation, apoptosis, and cell cycle decreased (Fig. 3b), demonstrating the potential of RNA editing tools in protein phosphorylation and dephosphorylation studies.

Here, we found higher levels of A-to-I and C-to-U RNA off-target SNPs in this study than was previously reported [5], especially for the dPspCas13b-RESCUE-NES system. In this study, we collected the top 20% of GFP-positive cells by FACS to examine the number of off-target events. This analysis method differs from the Abudayyeh et al. study [5], in which population cells were collected for off-target statistical analysis. This may be the main reason for the difference. The eRESCUE (dPspCas13b-RESCUE-NES) system improves the A-to-I and C-to-U RNA base editing efficiency. At the same time, the eRESCUE system reveals many RNA off-targets, which indicates that further optimisation is still needed. The RESCUE-S system, which is a safer variant, was also previously described by Abudayyeh et al. [5]. For this system, S375A of ADAR2 was fused with dRanCas13b. The RESCUE-S system significantly decreased RNA off-target events [5] and was therefore given the name eRESCUE-S as the safer (S) variant. S375A of ADAR2 was also fused with dPspCas13b, which may be more specific than the eRESCUE system. Some conditionally induced systems may be used to regulate the dCas13b and ADAR2 protein effective time; this could further reduce the RNA off-target events.

## Conclusions

In summary, we developed an efficient RNA base editor, dPspCas13b-RESCUE-NES, providing a potentially useful tool for biomedical research and genetic disease. Despite its high efficiency, however, higher numbers of off-target events were detected with the dPspCas13b-RESCUE-NES system relative to the control, given that further optimisation is still needed.

## Supplementary Information

**Additional file 1.Table S1**. Guide sequences used for endogenous gene editing. **Table S2**. Primers used in this study. **Table S3**. Q-PCR primers used in this study.Table S3. Q-PCR primers used in this study. **Table S4**. RNA base editor expression vectors.

## Data Availability

Graphpad Prism 8 is an open source collaborative initiative available in the Graphpad repository (https://www.graphpad.com/). Image J is an open source collaborative initiative available in the softonic repository (https://imagej.en.softonic.com/). EditR is an open source collaborative initiative available in the web (https://moriaritylab.shinyapps.io/editr_v10/). The RNA-seq data will be deposited at NCBI Bioproject (PRJNA656653).

## Abbreviations

REPAIR: RNA editing for programmable A-to-I replacement
RESCUE: RNA editing for specific C-to-U exchange
A-to-I: Adenosine-to-inosine
C-to-U: Cytidine-to-uridine
ADAR2: Adenosine deaminase acting on RNA type 2
*IKKβ*: Inhibitor kappa B kinase β
NES: Nuclear export sequence
FACS: Fluorescence activating cell sorter
