# Supplementary material for: Developing PspCas13b-based enhanced RESCUE system, eRESCUE, with efficient RNA base editing
Source: Cell Commun Signal. 2021 Aug 11;19:84. doi: 10.1186/s12964-021-00716-z (PMC8356384; doi:10.1186/s12964-021-00716-z)
Supplement: Supplementary file 2 — Additional file 1.Table S1. Guide sequences used for endogenous gene editing. Table S2. Primers used in this study. Table S3. Q-PCR primers used in this study.Table S3. Q-PCR primers used in this study. Table S4. RNA base editor expression vectors. [file 12964_2021_716_MOESM2_ESM.docx]

**Table S1. Guide sequences used for endogenous gene editing**

| Targeted gene | dPsp/RanCas13b-RESCUE-NES | Motif | Base flip | Spacer sequences |
| --- | --- | --- | --- | --- |
| *CTNNB1* | dPspCas13b-RESCUE-NES | UCC | C/20 | TGGTGCCACTACCACAGCTCCTTCTCTGAG |
| *CTNNB1* | dPspCas13b-RESCUE-NES | UAC | A/11 | TGGTGCCACTACCACAGCTCCTTCTCTGAG |
| *KRAS* site1 | dPspCas13b-RESCUE-NES | ACG | C/20 | TTCAGAATCATTTTGTGGACGAATATGATC |
| *KRAS* site1 | dPspCas13b-RESCUE-NES | UAU | A/25 | TTCAGAATCATTTTGTGGACGAATATGATC |
| *KRAS* site2 | dPspCas13b-RESCUE-NES | UCG | C/24 | GAAACCTGTCTCTTGGATATTCTCGACACA |
| *KRAS* site2 | dPspCas13b-RESCUE-NES | UAU | A/19 | GAAACCTGTCTCTTGGATATTCTCGACACA |
| *NF2* | dPspCas13b-RESCUE-NES | ACG | C/24 | TCTCAAGAGGAAGCAACCCAAGACGTTCAC |
| *NF2* | dPspCas13b-RESCUE-NES | AAG | A/12 | TCTCAAGAGGAAGCAACCCAAGACGTTCAC |
| *NRAS* | dPspCas13b-RESCUE-NES | UCC | C/20 | GGAAAAGCGCACTGACAATCCAGCTAATCC |
| *NFKB1* | dPspCas13b-RESCUE-NES | UCA | C/17 | TAATCCAGAAGTATTTCAACCACAGATGGC |
| *NFKB1* | dPspCas13b-RESCUE-NES | UAU | A/13 | TAATCCAGAAGTATTTCAACCACAGATGGC |
| *AHI1* | dPspCas13b-RESCUE-NES | GAU | A/24 | TATGAAAGAAACTACAAGTGATGATCCCGA |
| *AHI1* | dPspCas13b-RESCUE-NES | GCU | C/22 | CAAGTGATGATGTAAGTGCTGCTAACACTA |
| *APC* | dPspCas13b-RESCUE-NES | UAA | A/22 | GAGCTTAACTTAGATAGCAGTAATTTCCCT |
| *BMPR2* | dPspCas13b-RESCUE-NES | UCG | C/21 | TGGCCCAGGGATGACTTCCTCGCTGCAGCG |
| *DMD* | dPspCas13b-RESCUE-NES | UAU | A/23 | CAACGTCCCTCTCTGCGTGGATATGTGTCT |
| *DNAH5* | dPspCas13b-RESCUE-NES | UAG | A/23 | AGGAGGTGAGTTTTAACATGTTAGATGCGG |
| *IL2RG* | dPspCas13b-RESCUE-NES | GCA | C/21 | ACTGTTTGGAGCACTTGGTGCAGTACCGGA |
| *SCN9A* | dPspCas13b-RESCUE-NES | UAU | A/23 | CAATGCCACACCTGCTTTATATATGCTTTC |
| *SCN9A* | dPspCas13b-RESCUE-NES | UCU | C/24 | TTTCAGTCCTCTAAGAAGAATATCTATTAA |
| *TARDBP* | dPspCas13b-RESCUE-NES | UAG | A/23 | TAATGTCACAGCGACATATGATAGATGGAC |
| *TARDBP* | dPspCas13b-RESCUE-NES | UCU | C/23 | TATGGTGCAGGTCAAGAAAGATCTTAAGAC |
| *UBE3A* | dPspCas13b-RESCUE-NES | UAU | A/25 | GCTTCCTGTCCAACTTTTCTTCGTATGGAT |
| *CTNNB1* | dRan Cas13b-RESCUE-NES | UCC | C/20 | TGGTGCCACTACCACAGCTCCTTCTCTGAG |
| *CTNNB1* | dRan Cas13b-RESCUE-NES | UAC | A/11 | TGGTGCCACTACCACAGCTCCTTCTCTGAG |
| *KRAS* site1 | dRan Cas13b-RESCUE-NES | ACG | C/20 | TTCAGAATCATTTTGTGGACGAATATGATC |
| *KRAS* site1 | dRan Cas13b-RESCUE-NES | UAU | A/25 | TTCAGAATCATTTTGTGGACGAATATGATC |
| *KRAS* site2 | dRan Cas13b-RESCUE-NES | UCG | C/24 | GAAACCTGTCTCTTGGATATTCTCGACACA |
| *KRAS* site2 | dRan Cas13b-RESCUE-NES | UAU | A/19 | GAAACCTGTCTCTTGGATATTCTCGACACA |
| *NF2* | dRan Cas13b-RESCUE-NES | ACG | C/24 | TCTCAAGAGGAAGCAACCCAAGACGTTCAC |
| *NF2* | dRan Cas13b-RESCUE-NES | AAG | A/12 | TCTCAAGAGGAAGCAACCCAAGACGTTCAC |
| *NRAS* | dRan Cas13b-RESCUE-NES | UCC | C/20 | GGAAAAGCGCACTGACAATCCAGCTAATCC |
| *NFKB1* | dRan Cas13b-RESCUE-NES | UCA | C/17 | TAATCCAGAAGTATTTCAACCACAGATGGC |
| *NFKB1* | dRan Cas13b-RESCUE-NES | UAU | A/13 | TAATCCAGAAGTATTTCAACCACAGATGGC |
| *AHI1* | dRan Cas13b-RESCUE-NES | GAU | A/24 | TATGAAAGAAACTACAAGTGATGATCCCGA |
| *AHI1* | dRan Cas13b-RESCUE-NES | GCU | C/22 | CAAGTGATGATGTAAGTGCTGCTAACACTA |
| *BMPR2* | dRan Cas13b-RESCUE-NES | UCG | C/21 | TGGCCCAGGGATGACTTCCTCGCTGCAGCG |
| *DMD* | dRan Cas13b-RESCUE-NES | UAU | A/23 | CAACGTCCCTCTCTGCGTGGATATGTGTCT |
| *DNAH5* | dRan Cas13b-RESCUE-NES | UAG | A/23 | AGGAGGTGAGTTTTAACATGTTAGATGCGG |
| *IL2RG* | dRan Cas13b-RESCUE-NES | GCA | C/21 | ACTGTTTGGAGCACTTGGTGCAGTACCGGA |
| *SCN9A* | dRan Cas13b-RESCUE-NES | UAU | A/23 | CAATGCCACACCTGCTTTATATATGCTTTC |
| *SCN9A* | dRan Cas13b-RESCUE-NES | UCU | C/24 | TTTCAGTCCTCTAAGAAGAATATCTATTAA |
| *TARDBP* | dRan Cas13b-RESCUE-NES | UAG | A/23 | TAATGTCACAGCGACATATGATAGATGGAC |
| *TARDBP* | dRan Cas13b-RESCUE-NES | UCU | C/23 | TATGGTGCAGGTCAAGAAAGATCTTAAGAC |
| *UBE3A* | dRan Cas13b-RESCUE-NES | UAU | A/25 | GCTTCCTGTCCAACTTTTCTTCGTATGGAT |
| *IKKβ* | dPspCas13b-RESCUE-NES | CAG | A/22 | GCCAAGGAGCTGGATCAGGGCAGTCTTTGC |
| *IKKβ* | dPspCas13b-RESCUE-NES | CAG | A/24 | ATGCCAAGGAGCTGGATCAGGGCAGTCTTT |
| *IKKβ* | dPspCas13b-RESCUE-NES | CAG | A/26 | ATATGCCAAGGAGCTGGATCAGGGCAGTCT |

**Table S2. Primers used in this study.**

| Targeted gene | PCR primer sequences (5’-3’) |
| --- | --- |
| *CTNNB1* | F: CTTGGCTGTCTTTCAGATTTGAC; R: ACTCTCTTTTCTTCACCACAAC |
| *KRAS* site1/2 | F: GGCTCGGCCAGTACTCCCGG; R: GTCCTCATGTACTGGTCCCTCA |
| *NF2* | F: AAAGGGCTCAGAGTGCAGGC; R: CTCCTGAACCAGCTCCTCTT |
| *NRAS* | F: ATGGCGGTTCCGGGGTCTCC; R: GAAGCCTTCGCCTGTCCTCA |
| *NFKB1* | F: TTAGGAGGGAGAGCCCACCC; R: AGTGCAGATCCCATCCTCAC |
| *AHI1* | F: GCTGAGAGATGCCTACAGCT; R: CTGGCTGTGGCTTTGTATGT |
| *APC* | F: GCAAGTTGAGGCACTGAAGA; R: CAGCAAGAAGCAATGACCTC |
| *BMPR2* | F: GGAATTTCTGCAGCGGCATG; R: GCCATAGCAGGTGCTACCTT |
| *DMD* | F: CTCCGAAGACTGCAGAAGGC; R: TACTGCCCCCAAAGGATGCA |
| *DNAH5* | F: CTTGGCTCTCTAGGAGGGGT; R: CAAGTAGTCCGTAGGTTCCT |
| *IL2RG* | F: AGCCTACCAACCTCACTCTG; R: TTCACTCCAATGCTGAGCAC |
| *SCN9A* | F: CCCCAAAGCCAAGCAGTGAC; R: CAGTTCCACGGGTCACGAAG |
| *TARDBP* | F: ATGCTGGCTGGGGAAATCTG; R: CAAAGGCCCTGAATGGCTTG |
| *UBE3A* | F: GGCGACGACAGATCAGGAGA; R: CTTTCCGGAAGCTCTGTACC |
| *IKKβ* | F: TGCTGGCCATGGAGTACTGC; R: CACCTCACTCTTCTGCCGCA |

**Table S3. Q-PCR primers used in this study.**

| Detected gene | Q-PCR primer sequences (5’-3’) |
| --- | --- |
| *P53* | F: CAACGTTCTGTCCCCCTTGC; R: GTGTAGGAGCTGCTGGTGCA |
| *GADD45* | F: AGCTAGCTGATGACAGTGCA; R: GCAGTCCTGTGCATTGCATG |
| *c-Myc* | F: AGAATGTGGAGTGAACCCAG; R: TTGTCACTAAGGGATGGGGC |
| *cyclin D1* | F: ATGGAACACCAGCTCCTGTG; R: AGGACCTCCTTCTGCACACA |
| *Bcl2* | F: GGGATCGTTGCCTTATGCAT; R: GGATCTTTATTTCATGAGGCACG |
| *NF-kB* | F: TCTTCCTTCTCCAGCCGGCA; R: CAGGCCTTCCCAAATATGGA |
| *JNK1* | F: GGTTACCTCAGATTCCCCTG; R: GCTAGGTGGTGGAGAAACAG |
| *TRAF1* | F: AGTGACTTCTCCTTGAGGTC; R: GGAGCAGGAATTACCCTTTG |
| *TRAF2* | F: TACTGCTCCTTCTGCCTGGC; R: CGGGCAGCATTATCTGGGAA |
| *β-actin* (Human) | F: CTCCATCCTGGCCTCGCTGT; R: GCTGTCACCTTCACCGTTCC |
| *GAPDH* (Human) | F: AGAAGGCTGGGGCTCATTTG; R: AGGGGCCATCCACAGTCTTC |

**Table S4. RNA base editor expression vectors.**

NLS/NES, dCas13, and ADAR2 dd are highlighted in red, blue, and green, respectively. The start and stop codons are capitalized.

| RNA base editors’ sequences |
| --- |
| dRanCas13b-RESCUE-NES:  ATGgagaagcccctgctgcctaacgtgtataccctgaagcacaagttcttttggggcgccttcctgaatatcgcccggcacaacgcctttatcaccatctgccacatcaatgagcagctgggcctgaaaacccccagcaacgacgataagatcgtggacgtggtgtgcgagacatggaacaatatcctgaacaatgaccacgatctgctgaagaagtcccagctgaccgagctgatcctgaagcacttcccttttctgacagccatgtgctaccacccccctaagaaggagggcaagaagaagggccaccagaaggagcagcagaaggagaaggagagcgaggcacagtcccaggcagaggccctgaacccatccaagctgatcgaggccctggagatcctggtgaatcagctgcactctctggcaaactactatagcgccataagcacaagaagcccgacgccgagaaggatatcttcaagcacctgtataaggccttcgacgcctctctgagaatggtgaaggaggattataaggcccacttcaccgtgaatctgacaagggactttgcccacctgaaccgcaagggcaagaacaagcaggacaatcccgacttcaacagatacagattcgagaaggatggcttctttaccgagtctggcctgctgttctttacaaatctgtttctggacaagagggatgcctattggatgctgaagaaggtgtccggcttcaaggcctctcacaagcagcgcgagaagatgaccacagaggtgttttgcaggagccgcatcctgctgcccaagctgaggctggagtcccgctacgaccacaaccagatgctgctggatatgctgtctgagctgagcagatgtcctaagctgctgtatgagaagctgagcgaggagaataagaagcacttccaggtggaggccgacggctttctggatgagatcgaggaggagcagaacccattcaaggacaccctgatccggcaccaggatagattcccctactttgccctgaggtatctggacctgaatgagtccttcaagtctatccgctttcaggtggatctgggcacataccactattgtatctacgacaagaagatcggcgatgagcaggagaagaggcacctgacccgcacactgctgtccttcggccggctgcaggactttaccgagatcaacagaccccaggagtggaaggccctgaccaaggacctggattacaaggagacatccaatcagcctttcatctctaagaccacaccacactatcacatcaccgacaacaagatcggctttcggctgggcacaagcaaggagctgtacccctccctggagatcaaggatggcgccaatagaatcgccaagtacccttataactccggcttcgtggcccacgcctttatctctgtgcacgagctgctgcctctgatgttctaccagcacctgaccggcaagagcgaggacctgctgaaggagacagtgcggcacatccagagaatctataaggacttcgaggaggagcggatcaataccatcgaggatctggagaaggcaaaccagggcagactgccactgggagcctttcccaagcagatgctgggcctgctgcagaataagcagcctgatctgtccgagaaggccaagatcaagatcgagaagctgatcgccgagacaaagctgctgtctcacaggctgaacacaaagctgaagagctccccaaagctgggcaagcggagagagaagctgatcaagacaggcgtgctggccgactggctggtgaaggatttcatgcgctttcagcccgtggcctacgacgcccagaatcagcctatcaagtctagcaaggccaactccaccgagttctggtttatcaggcgcgccctggccctgtacggaggagagaagaataggctggagggctatttcaagcagaccaacctgatcggcaacacaaatccacaccccttcctgaacaagtttaattggaaggcctgcaggaatctggtggatttctaccagcagtatctggagcagcgcgagaagtttctggaggccatcaagaaccagccatgggagccctaccagtattgcctgctgctgaagatccctaaggagaacaggaagaatctggtgaagggatgggagcagggaggaatctctctgccacggggcctgtttaccgaggccatcagagagacactgtctgaggacctgatgctgagcaagccaatccgcaaggagatcaagaagcacggccgggtgggcttcatcagcagagccatcaccctgtactttaaggagaagtatcaggataagcaccagagcttctacaatctgtcctataagctggaggcaaaggcaccactgctgaagcgggaggagcactacgagtattggcagcagaacaagcctcagtctccaaccgagagccagaggctggagctgcacacaagcgaccgctggaaggattacctgctgtataagagatggcagcacctggagaagaagctgcggctgtaccggaatcaggacgtgatgctgtggctgatgaccctggagctgacaaagaaccacttcaaggagctgaacctgaattatcaccagctgaagctggagaatctggccgtgaacgtgcaggaggccgatgccaagctgaaccctctgaatcagaccctgcccatggtgctgcctgtgaaggtgtaccctgccacagcctttggcgaggtgcagtaccacaagaccccaatcaggacagtgtatatccgcgaggagcacaccaaggccctgaagatgggcaacttcaaggccctggtgaaggaccggagactgaatggcctgttctcttttatcaaggaggagaacgatacacagaagcacccaatcagccagctgcggctgaggcgcgagctggagatctaccagtctctgagagtggacgccttcaaggagacactgagcctggaggagaagctgctgaataagcacacatccctgtcctctctggagaacgagtttagggccctgctggaggagtggaagaaggagtatgccgccagctccatggtgacagacgagcacatcgccttcatcgccagcgtggccaatgccttctgcgccaaccagtaccccttctacaaggaggccctgcacgcccctatcccactgttcaccgtggcccagcccaccacagaggagaaggatggcctgggaatcgcagaggccctgctgaaggtgctgagggagtactgtgagatcgtgaagtcccagatcggatccCTTCAACTGCCTCCACTTGAAAGACTGACACTGggatcccagctgcatttaccgcaggttttagctgacgctgtctcacgcctggtcataggtaagtttggtgacctgaccgacaacttctcctcccctcacgctcgcagaataggtctggctggagtcgtcatgacaacaggcacagatgttaaagatgccaaggtgatatgtgtttctacaggagcaaaatgtattaatggtgaatacctaagtgatcgtggccttgcattaaatgactgccatgcagaaatagtatctcggagatccttgctcagatttctttatacacaacttgagctttacttaaataacgaggatgatcaaaaaagatccatctttcagaaatcagagcgaggggggtttaggctgaaggagaatatacagtttcatctgtacatcagcacctctccctgtggagatgccagaatcttctcaccacatgaggcaatcctggaagaaccagcagatagacacccaaatcgtaaagcaagaggacagctacggaccaaaatagaggctggtcaggggacgattccagtgcgcaacaatgcgagcatccaaacgtgggacggggtgctgcaaggggagcggctgctcaccatgtcctgcagtgacaagattgcacgctggaacgtggtgggcatccagggatcactgctcagcattttcgtggagcccatttacttctcgagcatcatcctgggcagcctttaccacggggaccacctttccagggccatgtaccagcggatctccaacatagaggacctgccacctctctacaccctcaacaagcctttgctcacaggcatcagcaatgcagaagcacggcagccagggaaggcccccatattcagtgtcaactggacggtaggcgactccgctattgaggtcatcaacgccacgactgggaagggagagctgggccgcgcgtcccgcctgtgtaagcacgcgttgtactgtcgctggatgcgtgtgcacggcaaggttccctcccacttactacgctccaagattaccaagcccaacgtgtaccatgagacaaagctggcggcaaaggagtaccaggccgccaaggcgcgtctgttcacagccttcatcaaggcggggctgggggcctgggtggagaagcccaccgagcaggaccagttctcactcacgTAA |
| dPspCas13b-RESCUE-NES:  ATGaacatccccgctctggtggaaaaccagaagaagtactttggcacctacagcgtgatggccatgctgaacgctcagaccgtgctggaccacatccagaaggtggccgatattgagggcgagcagaacgagaacaacgagaatctgtggtttcaccccgtgatgagccacctgtacaacgccaagaacggctacgacaagcagcccgagaaaaccatgttcatcatcgagcggctgcagagctacttcccattcctgaagatcatggccgagaaccagagagagtacagcaacggcaagtacaagcagaaccgcgtggaagtgaacagcaacgacatcttcgaggtgctgaagcgcgccttcggcgtgctgaagatgtacagggacctgaccaacgcatacaagacctacgaggaaaagctgaacgacggctgcgagttcctgaccagcacagagcaacctctgagcggcatgatcaacaactactacacagtggccctgcggaacatgaacgagagatacggctacaagacagaggacctggccttcatccaggacaagcggttcaagttcgtgaaggacgcctacggcaagaaaaagtcccaagtgaataccggattcttcctgagcctgcaggactacaacggcgacacacagaagaagctgcacctgagcggagtgggaatcgccctgctgatctgcctgttcctggacaagcagtacatcaacatctttctgagcaggctgcccatcttctccagctacaatgcccagagcgaggaacggcggatcatcatcagatccttcggcatcaacagcatcaagctgcccaaggaccggatccacagcgagaagtccaacaagagcgtggccatggatatgctcaacgaagtgaagcggtgccccgacgagctgttcacaacactgtctgccgagaagcagtcccggttcagaatcatcagcgacgaccacaatgaagtgctgatgaagcggagcagcgacagattcgtgcctctgctgctgcagtatatcgattacggcaagctgttcgaccacatcaggttccacgtgaacatgggcaagctgagatacctgctgaaggccgacaagacctgcatcgacggccagaccagagtcagagtgatcgagcagcccctgaacggcttcggcagactggaagaggccgagacaatgcggaagcaagagaacggcaccttcggcaacagcggcatccggatcagagacttcgagaacatgaagcgggacgacgccaatcctgccaactatccctacatcgtggacacctacacacactacatcctggaaaacaacaaggtcgagatgtttatcaacgacaaagaggacagcgccccactgctgcccgtgatcgaggatgatagatacgtggtcaagacaatccccagctgccggatgagcaccctggaaattccagccatggccttccacatgtttctgttcggcagcaagaaaaccgagaagctgatcgtggacgtgcacaaccggtacaagagactgttccaggccatgcagaaagaagaagtgaccgccgagaatatcgccagcttcggaatcgccgagagcgacctgcctcagaagatcctggatctgatcagcggcaatgcccacggcaaggatgtggacgccttcatcagactgaccgtggacgacatgctgaccgacaccgagcggagaatcaagagattcaaggacgaccggaagtccattcggagcgccgacaacaagatgggaaagagaggcttcaagcagatctccacaggcaagctggccgacttcctggccaaggacatcgtgctgtttcagcccagcgtgaacgatggcgagaacaagatcaccggcctgaactaccggatcatgcagagcgccattgccgtgtacgatagcggcgacgattacgaggccaagcagcagttcaagctgatgttcgagaaggcccggctgatcggcaagggcacaacagagcctcatccatttctgtacaaggtgttcgcccgcagcatccccgccaatgccgtcgagttctacgagcgctacctgatcgagcggaagttctacctgaccggcctgtccaacgagatcaagaaaggcaacagagtggatgtgcccttcatccggcgggaccagaacaagtggaaaacacccgccatgaagaccctgggcagaatctacagcgaggatctgcccgtggaactgcccagacagatgttcgacaatgagatcaagtcccacctgaagtccctgccacagatggaaggcatcgacttcaacaatgccaacgtgacctatctgatcgccgagtacatgaagagagtgctggacgacgacttccagaccttctaccagtggaaccgcaactaccggtacatggacatgcttaagggcgagtacgacagaaagggctccctgcagcactgcttcaccagcgtggaagagagagaaggcctctggaaagagcgggcctccagaacagagcggtacagaaagcaggccagcaacaagatccgcagcaaccggcagatgagaaacgccagcagcgaagagatcgagacaatcctggataagcggctgagcaacagccggaacgagtaccagaaaagcgagaaagtgatccggcgctacagagtgcaggatgccctgctgtttctgctggccaaaaagaccctgaccgaactggccgatttcgacggcgagaggttcaaactgaaagaaatcatgcccgacgccgagaagggaatcctgagcgagatcatgcccatgagcttcaccttcgagaaaggcggcaagaagtacaccatcaccagcgagggcatgaagctgaagaactacggcgacttctttgtgctggctagcgacaagaggatcggcaacctgctggaactcgtgggcagcgacatcgtgtccaaagaggatggatccCTTCAACTGCCTCCACTTGAAAGACTGACACTGggatcccagctgcatttaccgcaggttttagctgacgctgtctcacgcctggtcataggtaagtttggtgacctgaccgacaacttctcctcccctcacgctcgcagaataggtctggctggagtcgtcatgacaacaggcacagatgttaaagatgccaaggtgatatgtgtttctacaggagcaaaatgtattaatggtgaatacctaagtgatcgtggccttgcattaaatgactgccatgcagaaatagtatctcggagatccttgctcagatttctttatacacaacttgagctttacttaaataacgaggatgatcaaaaaagatccatctttcagaaatcagagcgaggggggtttaggctgaaggagaatatacagtttcatctgtacatcagcacctctccctgtggagatgccagaatcttctcaccacatgaggcaatcctggaagaaccagcagatagacacccaaatcgtaaagcaagaggacagctacggaccaaaatagaggctggtcaggggacgattccagtgcgcaacaatgcgagcatccaaacgtgggacggggtgctgcaaggggagcggctgctcaccatgtcctgcagtgacaagattgcacgctggaacgtggtgggcatccagggatcactgctcagcattttcgtggagcccatttacttctcgagcatcatcctgggcagcctttaccacggggaccacctttccagggccatgtaccagcggatctccaacatagaggacctgccacctctctacaccctcaacaagcctttgctcacaggcatcagcaatgcagaagcacggcagccagggaaggcccccatattcagtgtcaactggacggtaggcgactccgctattgaggtcatcaacgccacgactgggaagggagagctgggccgcgcgtcccgcctgtgtaagcacgcgttgtactgtcgctggatgcgtgtgcacggcaaggttccctcccacttactacgctccaagattaccaagcccaacgtgtaccatgagacaaagctggcggcaaaggagtaccaggccgccaaggcgcgtctgttcacagccttcatcaaggcggggctgggggcctgggtggagaagcccaccgagcaggaccagttctcactcacgTAA |
